# Bard1‐Mediated Regulation of Hnrnpa2b1 Ubiquitination and Protein Stability Contributes to Neuronal Ferroptosis and Cognitive Dysfunction Following Ischemic Stroke

**DOI:** 10.1002/cns.71074

**Published:** 2026-08-10

**Authors:** Tuming Li, Tianyi Rong, Zuoting Shen, Zhenyu Wei, Deyan Chen, Ping Zhong, Li Cao

**Affiliations:** ^1^ Department of Neurology Suzhou Hospital of Anhui Medical University, Suzhou Municipal Hospital of Anhui Province Suzhou Anhui People's Republic of China; ^2^ Department of Neurology Shidong Hospital Shanghai People's Republic of China; ^3^ Department of Neurology Shanghai Sixth People's Hospital Affiliated to Shanghai Jiao Tong University School of Medicine Shanghai People's Republic of China; ^4^ Department of Genetics and Rare Diseases Shanghai Sixth People's Hospital Affiliated to Shanghai Jiao Tong University School of Medicine Shanghai People's Republic of China; ^5^ Shanghai Neurological Rare Disease Biobank and Precision Diagnostic Technical Service Platform Shanghai People's Republic of China; ^6^ Neurological Disorder Center Haikou Orthopedic and Diabetes Hospital of Shanghai Sixth People's Hospital Haikou People's Republic of China

**Keywords:** Bard1, Ferroptosis, Hnrnpa2b1, post‐stroke cognitive impairment, *Sptbn2*

## Abstract

**Aims:**

N6‐methyladenosine (m^6^A) facilitates functional recovery following ischemic stroke (IS). This study investigated the role of *Sptbn2* in post‐stroke cognitive impairment (PSCI) and the mechanisms regarding m^6^A.

**Methods:**

HT‐22 cell damage and ferroptosis were analyzed following OGD exposure. pMCAO surgery was performed to establish an IS mouse model. We assessed neurological deficits and cognitive impairment in mice using the mNSS, adhesive removal test, rotarod test, novel object recognition test, and Y‐maze test. Adeno‐associated viral vectors with overexpression of *Sptbn2* combined with pMCAO surgery were used to analyze cognitive dysfunction and ferroptosis.

**Results:**

*Sptbn2* was reduced in neurons of PSCI mice. *Sptbn2* overexpression alleviated ferroptosis‐induced neuronal damage by promoting the membrane translocation of Slc7a11. Hnrnpa2b1 promoted *Sptbn2* stability through an m^6^A‐related mechanism. Knockdown of *Sptbn2* reversed the mitigation of ferroptosis by *Hnrnpa2b1* and exacerbated the neuronal injury. Under OGD, *Bard1* knockdown reduced the Hnrnpa2b1 ubiquitination, slowed Hnrnpa2b1 degradation, and restored Sptbn2 expression. Knockdown of *Bard1* alleviated neuronal ferroptosis, thereby reducing the development of cognitive impairment in mice, a phenotype reversed by *Hnrnpa2b1* or *Sptbn2* knockdown.

**Conclusion:**

In IS, Bard1‐associated regulation of Hnrnpa2b1 ubiquitination is accompanied by reduced Sptbn2 expression and impaired Slc7a11 membrane translocation, and is involved in neuronal ferroptosis‐related damage.

## Graphical abstract

Bard1‐mediated ubiquitination of Hnrnpa2b1 in mice with PSCI destabilizes *Sptbn2*, which prevents the membrane translocation of Slc7a11 and reduces cystine uptake. This disruption of antioxidant defense triggers ferroptosis in hippocampal neurons, highlighting a novel molecular pathway linking post‐stroke cognitive impairment to ferroptosis of hippocampal neurons.

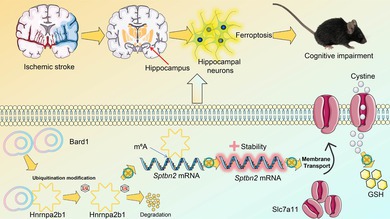

## 1 Introduction

Post‐stroke cognitive impairment (PSCI) occurs in approximately 60% of patients with stroke in the first year after onset [1]. There is an emerging consensus that the risk of PSCI is determined by the interplay of various factors, including modifiable and non‐modifiable risk factors, comorbidities, index stroke characteristics, features of acute infarct or hemorrhage, and the overall burden of pre‐existing brain injury [2]. The most prevalent deficits were observed in learning, verbal and figural episodic memory, constructive abilities, and selective attention, while deficits were identified across all cognitive domains [3]. Consequently, there is an imperative need for precise diagnosis, prompt intervention, and effective prevention of PSCI.

Spectrins are a class of cytoskeletal proteins that are widely expressed in the mammalian nervous system, and pathogenic variants in *SPTAN1*, *SPTBN1*, *SPTBN2*, and *SPTBN4*, four of the six genes encoding neuronal spectrins, have been associated with neurological disorders [4]. Among these, *SPTBN2* has been reported to interact with solute carrier family 7 member 11 (SLC7A11, also termed xCT), facilitating its membrane localization and suppressing ferroptosis in non‐small cell lung cancer [5]. Ferroptosis, characterized by lipid peroxidation and iron accumulation, has also been implicated in ischemic stroke (IS), and alterations in ferroptosis‐related genes, such as *GPX4* and *SLC7A11*, have been observed [6, 7]. This process is primarily triggered by the disruption of the cellular antioxidant system, particularly the system Xc^−^‐glutathione (GSH)‐GPX4 axis [8]. Slc7a11 plays a critical role in the import of cysteine, an essential precursor for GSH biosynthesis and antioxidant defense [9]. However, whether *Sptbn2*‐mediated membrane localization of Slc7a11 is involved in PSCI remains unknown.

*SPTBN2* has been reported to be regulated by METTL3‐mediated N6‐methyladenosine (m^6^A) modification in colorectal cancer [10]. m^6^A modification affects the processing, structure, localization, stability, degradation, and translation of RNAs and their functions, such as RNA‐protein and RNA–RNA interactions [11]. Generally, the regulation and function of m^6^A modification involve three types of proteins: methyltransferases (writers), RNA‐binding proteins (RBPs, readers), and demethylases (erasers) [12]. The RBP heterogeneous nuclear ribonucleoproteins A2/B1 (hnRNPA2B1) is a member of the hnRNP family that can recognize and bind specific RNA substrates and DNA motifs, which enables the modulation of their transcription, splicing, processing, transport, stability, and translation, and is involved in the development of neurodegenerative diseases [13]. Although it has recently been reported to be a key m^6^A regulator of IS [14], its specific role and mechanism in PSCI remain unknown. Therefore, this study aimed to evaluate the impact of Hnrnpa2b1/Sptbn2 on neuronal ferroptosis‐related damage in PSCI by subjecting mice to permanent middle cerebral artery occlusion (pMCAO) and mouse hippocampal HT‐22 neurons to oxygen–glucose deprivation (OGD).

## 2 Materials and Methods

### 2.1 RT‐qPCR

Total RNA was extracted from the cells using TRIzol (NR0002, Beijing Leagene Biotech Co. Ltd., Beijing, China). After quantifying RNA concentration and purity using a spectrophotometer, RNA was converted to cDNA using the First Strand cDNA Synthesis Kit (OriGene Technologies, Beijing, China). qPCR was performed using SensiMix SYBR Master Mix (QP100001, OriGene). The 2^−ΔΔCT^ method was used to evaluate relative gene expression. The results were normalized to the levels of the endogenous control, *Gapdh*. The primer sequences are listed in Table S1.

### 2.2 Statistics

All data were presented as mean ± standard deviation (SD), and statistical analyses were performed by GraphPad Prism 10.0.2 software (GraphPad, San Diego, CA, USA). Significance was assessed using the unpaired *t*‐test for comparisons between the two groups. Ordinary one‐way ANOVA and two‐way ANOVA, followed by Tukey's test, were used for comparisons of ≥ 3 groups. Results were considered statistically significant at *p* < 0.05.

More details can be found in the Supplementary Methods file.

## 3 Results

### 3.1 *Sptbn2* Overexpression in Hippocampal Neurons Alleviates OGD‐Induced Ferroptosis

We downloaded the transcriptomics sequencing data of HT‐22 cells after OGD treatment from GSE178997 in the GEO database and performed differential analysis (DESeq2) using the Hiplot tool (https://hiplot.com.cn/home/index.html). With Benjamini and Hochberg for *p*‐value correction, a screening threshold of adjusted *p*‐value < 0.01 and |LogFC| > 1 was set to screen for differentially expressed genes directly associated with ischemic‐like damage in neurons (Figure S1A). Given that the peripheral circulatory transcriptome of patients with IS reflects systemic transcriptional changes associated with the disease state, we used the GEO2R tool in the GEO database to analyze the transcriptomic alterations in the circulation of patients with IS and control patients in the GSE122709 and GSE140275 datasets (Figure S1B,C). Under the same filtering threshold, the intersection of differentially expressed genes across the three datasets yielded two molecules (Figure S1D): NEAT1 and SPTBN2. The role of NEAT1 in IS has been confirmed [15]. SPTBN2 was thus selected.

**FIGURE 1:**
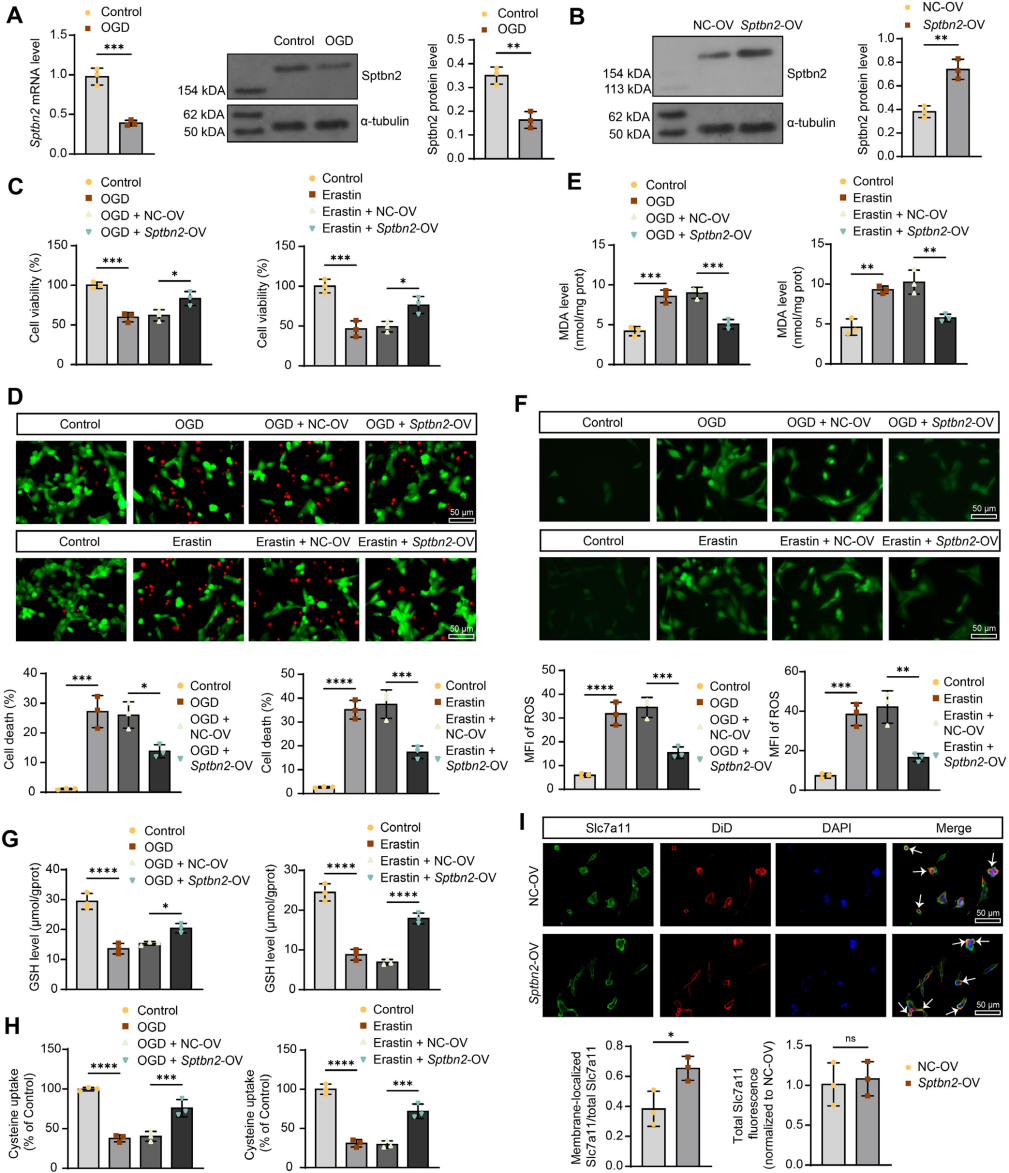
*Sptbn2* overexpression in hippocampal neurons alleviates OGD‐induced ferroptosis. (A) The mRNA and protein expression of Sptbn2 in HT‐22 cells with OGD was examined using RT‐qPCR and Western blot assay. (B) Sptbn2 expression after overexpression of *Sptbn2* in HT‐22 cells was examined using a Western blot assay. (C) Cell viability in HT‐22 cells treated with OGD or Erastin and after overexpression of *Sptbn2* was examined using the CCK‐8 assay. (D) Cell death in HT‐22 cells treated with OGD or Erastin and after overexpression of *Sptbn2* was examined using live‐dead cell staining. (E) MDA content in HT‐22 cells treated with OGD or Erastin and after overexpression of *Sptbn2* was examined using an MDA content assay kit. (F) ROS production in HT‐22 cells treated with OGD or Erastin and after overexpression of *Sptbn2* was examined using a DCFH‐DA probe. (G) GSH content in HT‐22 cells treated with OGD or Erastin, and after overexpression of *Sptbn2*, was examined using a GSH assay kit. (H) The cystine uptake capacity of HT‐22 cells treated with OGD or Erastin, and after overexpression of *Sptbn2*, was examined using a cystine uptake assay. (I) The membrane transport of Slc7a11 following *Sptbn2* overexpression in HT‐22 cells was examined using DiD staining combined with immunofluorescence analysis. The DiD‐positive regions of the cell membrane were designated as regions of interest (ROIs) for membrane localization, and the Slc7a11 fluorescence intensity within this ROI and the total Slc7a11 fluorescence intensity in the same cell were measured. The total Slc7a11 fluorescence intensity was also quantified. *n* = 3 independent biological replicates. Results are expressed as mean ± SD; * indicates *p* < 0.05; ** indicates *p* < 0.01; *** indicates *p* < 0.001; **** indicates *p* < 0.0001. A two‐tailed unpaired *t*‐test (A–B, I) and one‐way ANOVA (C–H) were used for statistical analysis, respectively.

Sptbn2 expression was reduced in OGD‐treated HT‐22 cells (Figure 1A). In this regard, we overexpressed *Sptbn2* in HT‐22 cells (Figure 1B), followed by OGD. Erastin, a ferroptosis inducer, was used as a positive control. Both OGD and Erastin decreased cell viability (Figure 1C) and increased cell death (Figure 1D), whereas *Sptbn2* overexpression partially alleviated cellular damage. MDA levels (Figure 1E) and ROS levels (Figure 1F) increased after OGD and Erastin treatment and were ameliorated after *Sptbn2* overexpression. The decrease in GSH content in cells induced by OGD treatment or Erastin was reversed after *Sptbn2* overexpression (Figure 1G). *Sptbn2* overexpression also alleviated the impaired cystine uptake caused by OGD or Erastin (Figure 1H). We used DiD to label the cell membranes and immunofluorescence to analyze the membrane translocation of Slc7a11. Following *Sptbn2* overexpression, the proportion of Slc7a11 fluorescence within DiD‐positive membrane regions was increased relative to the total Slc7a11 fluorescence, while *Sptbn2* overexpression did not significantly alter the total Slc7a11 fluorescence (Figure 1I).

To clarify whether the alleviation of ferroptosis by Sptbn2 was realized through the Xc‐system, we used RSL3, an inhibitor of GPX4, to treat the cells. Overexpression of *Sptbn2* did not alleviate cellular damage induced by RSL3 (Figure S1E,F), and MDA and ROS levels were not affected by *Sptbn2* overexpression (Figure S1G,H), suggesting that *Sptbn2* overexpression did not alleviate ferroptosis induced by GPX4 inhibition.

### 3.2 Fer‐1 Reverses OGD‐Induced Neuronal Damage Exacerbated by *Sptbn2* Knockdown

To verify whether the Sptbn2 deficiency exacerbated OGD‐induced neuronal damage through ferroptosis, we administered Fer‐1 in the context of *Sptbn2* knockdown (Figure S2A). *Sptbn2* knockdown further exacerbated the HT‐22 cell viability reduction (Figure S2B), increases in MDA (Figure S2C) and ROS levels (Figure S2D), and a decrease in GSH levels (Figure S2E) induced by OGD. Fer‐1 reversed the decreases in cell viability, the increases in MDA and ROS levels, and the decrease in GSH levels caused by *Sptbn2* knockdown.

### 3.3 Overexpression of *Sptbn2* in Neurons Alleviates Ferroptosis and PSCI in Mice

**FIGURE 2:**
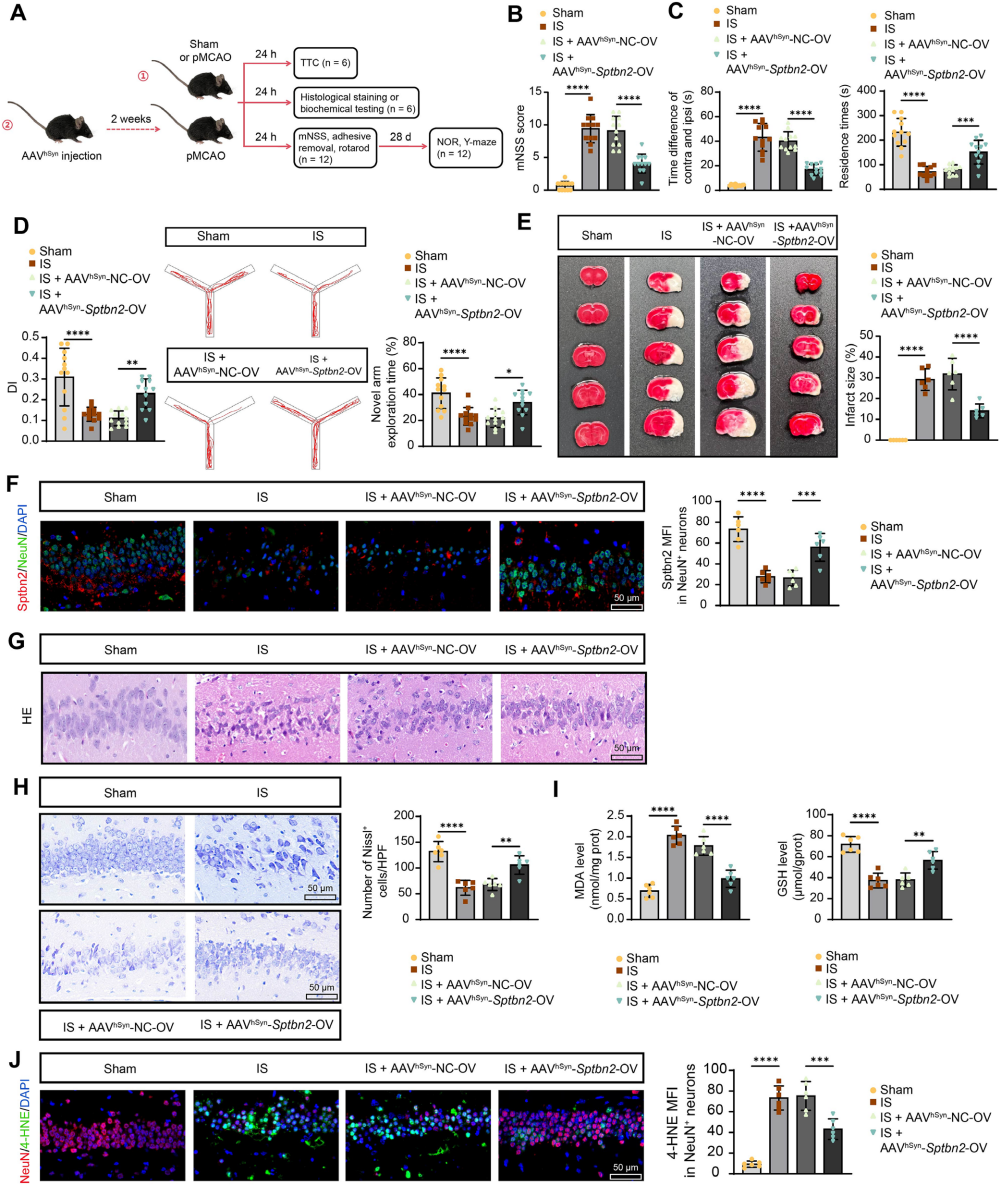
Overexpression of *Sptbn2* in neurons mitigates ferroptosis to alleviate behavioral deficits in IS mice. (A) Flow chart of pMCAO and AAV vector injection in mice. Twenty‐four h post‐surgery, tissue samples were collected for TTC staining (*n* = 6 independent biological replicates) and histological staining or biochemical assays (*n* = 6 independent biological replicates). Behavioral assessments (*n* = 12 independent biological replicates) were conducted on the same cohort both 24 h and 28 d post‐surgery. (B) Postoperative neurologic impairment assessed by mNSS scoring (*n* = 12 independent biological replicates). (C) The adhesive removal time between the ipsilateral and contralateral regions of the infarct in the adhesive removal test and the residence time of the mice on the rotarod in the rotarod test (*n* = 12 independent biological replicates). (D) DI in the novel object recognition and novel arm exploration time in the Y‐maze tests (*n* = 12 independent biological replicates). (E) The area of cerebral infarction [(area of the contralateral hemisphere—area of the ipsilateral non‐infarct region)/area of the contralateral hemisphere × 100%] of mice was evaluated using TTC staining (*n* = 6 independent biological replicates). (F) MFI of Sptbn2 in hippocampal neurons of the CA1 region of hippocampal tissue of mice was detected by dual‐labeling immunofluorescence (*n* = 6 independent biological replicates). (G) Neuropathological damage in the CA1 region of the hippocampal tissue of mice was detected by HE staining (*n* = 6 independent biological replicates). (H) The number of Nissl‐positive neurons in the CA1 area of hippocampal tissue of mice was detected by Nissl staining (*n* = 6 independent biological replicates). (I) MDA and GSH contents in hippocampal tissue homogenates from mice in each group (24 h after pMCAO) were measured by biochemical analysis (normalized to total protein concentration) (*n* = 6 independent biological replicates). (J) The MFI of 4‐HNE in NeuN‐positive neurons in the hippocampal CA1 region of mice in each group 24 h after pMCAO was detected by dual‐labeling immunofluorescence (*n* = 6 independent biological replicates). Results are expressed as mean ± SD; * indicates *p* < 0.05; ** indicates *p* < 0.01; *** indicates *p* < 0.001; **** indicates *p* < 0.0001. One‐way ANOVA (B–F, H–J) was used for statistical analysis.

We injected AAV vectors encapsulating overexpression of the *Sptbn2* plasmid (*Sptbn2*‐OV) into mouse hippocampal tissues, followed by pMCAO (Figure 2A). Approximately 24 h after surgery, the IS mice showed neurobehavioral deficits (Figure 2B). Adhesive removal and rotarod tests confirmed limb incoordination in the IS mice (Figure 2C). The analysis of cognitive dysfunction at 28 days post‐surgery showed that the discrimination index (DI) and exploration time of mice in the novel arm were lowered after pMCAO modeling (Figure 2D). PSCI was partially improved by *Sptbn2*‐OV.

Twenty‐four hours postoperatively, *Sptbn2*‐OV reduced the cerebral infarction area in mice (Figure 2E). The mean fluorescence intensity (MFI) of *Sptbn2* in hippocampal neurons was reduced in IS mice and increased after *Sptbn2*‐OV (Figure 2F). *Sptbn2*‐OV alleviated degeneration (Figure 2G) and loss of neurons (Figure 2H) in the CA1 area of the hippocampus of IS mice. The increase in MDA levels and decrease in GSH content in IS mice 24 h after surgery were ameliorated by *Sptbn2*‐OV (Figure 2I). In the CA1 region of the hippocampus 24 h after IS surgery in mice, the MFI of 4‐HNE in NeuN‐positive neurons was significantly elevated, whereas overexpression of *Sptbn2* partially reduced the MFI of 4‐HNE (Figure 2J).

The IS mice continued to exhibit elevated MDA levels and reduced GSH levels at 3 and 7 days post‐injury. By day 14, these changes had subsided compared to earlier time points but had not yet fully resolved. In contrast, *Sptbn2*‐OV reduced MDA levels to varying degrees and restored GSH levels at all time points (Figure S3A,B).

### 3.4 Hnrnpa2b1 Regulates the Stability of *Sptbn2*

We downloaded RBPs that bind to the presence of *Sptbn2* in *Mus musculus* from the RNAinter database (http://www.rnainter.org/) (Table S2). Genes related to IS were downloaded from the Genecards database (https://www.genecards.org/) using the keyword “ischemic stroke”. The intersection of the two lists revealed 34 possible candidates (Figure S4A). The protein–protein interaction (PPI) network was plotted in the STRING database (https://string‐db.org/), setting the minimum required interaction score as the highest confidence (0.900) (Figure S4B). Among the PPI networks centered on Hnrnpa2b1, Srsf1, Srsf2, and Srsf3, Hnrnpa2b1 had a higher relevance score associated with IS in the GeneCards database. Thus, we chose Hnrnpa2b1 for this study (Figure S4C). We obtained the mRNA sequence of *Sptbn2* from NCBI (NM_021287.2) and predicted the presence of m^6^A sites using the SRAMP tool (http://www.cuilab.cn/m6asiteapp/old), which showed that there were multiple m^6^A modification sites with very high confidence (Figure S4D). Combined with the significant positive correlation between *Hnrnpa2b1* and *Sptbn2* expression in normal hippocampal tissues analyzed in the GEPIA database (http://gepia.cancer‐pku.cn/index.html) (Figure S4E), we hypothesized that *Sptbn2* expression is regulated by the post‐transcriptional modification of Hnrnpa2b1.

The MFI of Hnrnpa2b1 was reduced in NeuN‐positive neurons in the CA1 region of the hippocampus in IS mice (Figure S5A). We also observed reduced expression of Hnrnpa2b1 in OGD‐treated HT‐22 cells (Figure S5B). MeRIP‐qPCR analysis showed that, compared with the corresponding control groups, the m^6^A enrichment of *Sptbn2* was significantly reduced in the hippocampal tissues of IS mice and in OGD‐treated HT‐22 cells (Figure S5C,D). To understand whether Hnrnpa2b1 regulates the mRNA stability of *Sptbn2*, we overexpressed Hnrnpa2b1 in HT‐22 cells (Figure S5E). *Hnrnpa2b1* overexpression induced *Sptbn2* expression (Figure S5F). OGD treatment decreased the stability of *Sptbn2*. Degradation of *Sptbn2* was partially slowed when we overexpressed *Hnrnpa2b1* (Figure S5G). SRAMP prediction results showed that multiple potential m^6^A sites in *Sptbn2* were distributed across the 3'UTR. Based on this, we constructed luciferase reporter vectors containing either the wild‐type (WT) *Sptbn2* 3'UTR sequence or sequences with predicted m6A site mutations. Overexpression of *Hnrnpa2b1* enhanced the luciferase activity of the WT vector, whereas this effect was lost following mutations at the predicted m6A sites (Figure S5H,I). RIP‐qPCR further demonstrated that, compared with IgG, *Sptbn2* mRNA was enriched in the Hnrnpa2b1 immunoprecipitation complex (Figure S5J).

### 3.5 Overexpression of *Hnrnpa2b1* Alleviates OGD‐Induced Neuronal Ferroptosis

**FIGURE 3:**
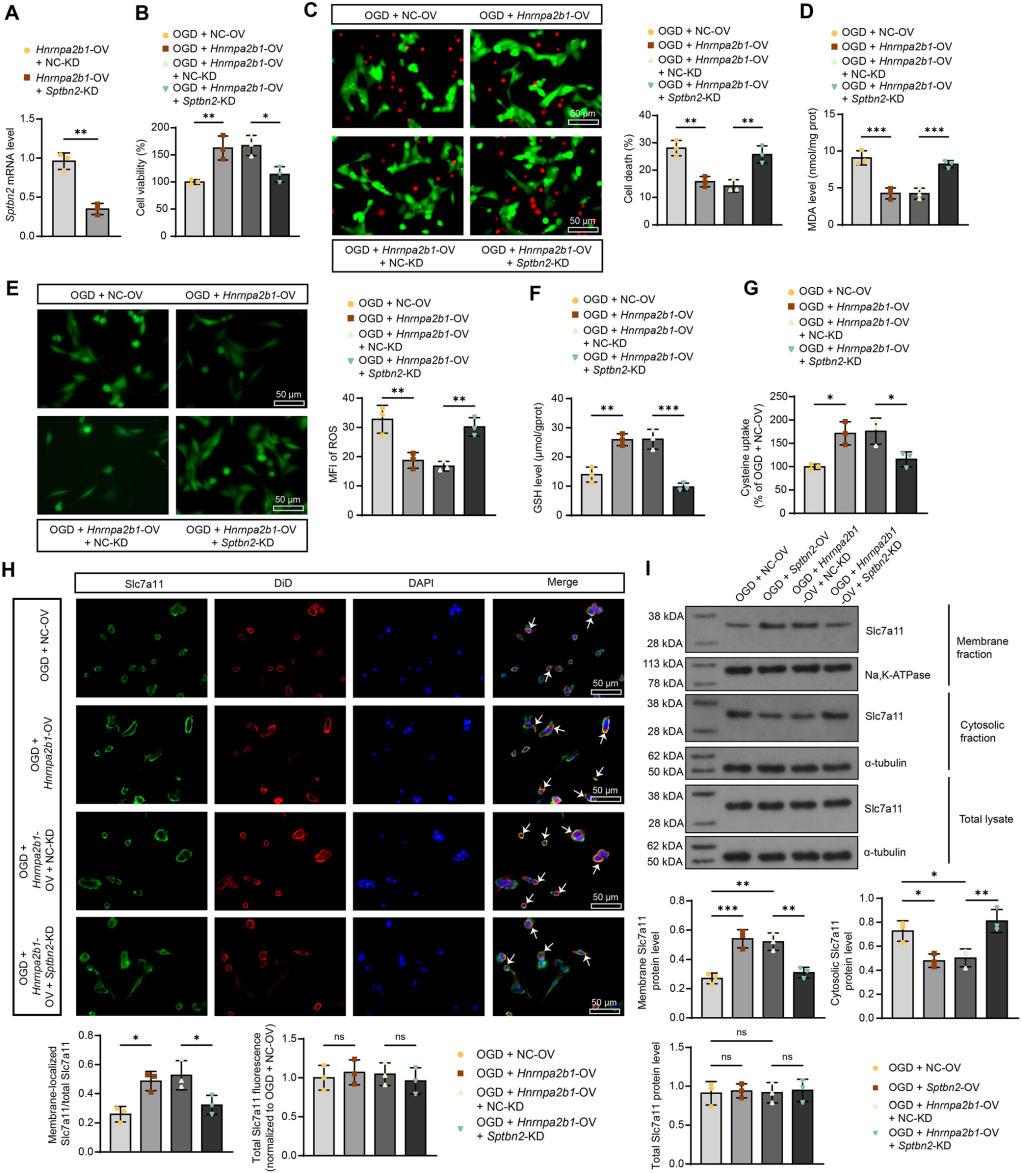
Overexpression of *Hnrnpa2b1* alleviates OGD‐induced ferroptosis, and knockdown of *Sptbn2* impairs this effect. (A) *Sptbn2* expression in HT‐22 cells transfected with *Hnrnpa2b1*‐OV + NC‐KD or *Hnrnpa2b1*‐OV + *Sptbn2*‐KD was examined using RT‐qPCR. (B) Cell viability in HT‐22 cells was examined using the CCK‐8 assay. (C) Cell death in HT‐22 cells was examined using live‐dead cell staining. (D) MDA content in HT‐22 cells was examined using an MDA content assay kit. (E) ROS production in HT‐22 cells was examined using a DCFH‐DA probe. (F) GSH content in HT‐22 cells was examined using a GSH assay kit. (G) The cystine uptake capacity of HT‐22 cells was examined using a cystine uptake assay. (H) The membrane transport of Slc7a11 in HT‐22 cells was examined using DiD staining combined with immunofluorescence analysis. The DiD‐positive regions of the cell membrane were designated as regions of interest (ROIs) for membrane localization, and the Slc7a11 fluorescence intensity within this ROI and the total Slc7a11 fluorescence intensity in the same cell were measured. The total Slc7a11 fluorescence intensity was also quantified. (I) The distribution of Slc7a11 in the membrane fraction, cytosolic fraction, and total protein in OGD‐treated HT‐22 cells was examined using membrane/cytosolic protein fractionation and Western blot analysis. *n* = 3 independent biological replicates. Results are expressed as mean ± SD; * indicates *p* < 0.05; ** indicates *p* < 0.01; *** indicates *p* < 0.001. A two‐tailed unpaired *t*‐test (A) and one‐way ANOVA (B–I) were used for statistical analysis, respectively.

Next, we knocked down *Sptbn2* (*Sptbn2*‐KD) based on the overexpression of *Hnrnpa2b1* (*Hnrnpa2b1*‐OV) in HT‐22 cells (Figure 3A). *Hnrnpa2b1*‐OV alleviated OGD‐induced cell injury (Figure 3B) and death (Figure 3C), which were attenuated by *Sptbn2*‐KD. In addition, the reduction in MDA (Figure 3D) and ROS levels (Figure 3E) in cells caused by *Hnrnpa2b1*‐OV was weakened by *Sptbn2*‐KD. In terms of the XC system‐mediated cystine uptake and GSH production, *Hnrnpa2b1*‐OV induced the restoration of GSH levels (Figure 3F) and increased cystine uptake (Figure 3G), whereas *Sptbn2*‐KD decreased the levels of both. *Hnrnpa2b1* overexpression‐induced Slc7a11 membrane transport was attenuated following *Sptbn2*‐KD, while the total Slc7a11 fluorescence intensity did not change significantly across all groups (Figure 3H). Subsequently, overexpression of either *Sptbn2* or *Hnrnpa2b1* significantly increased Slc7a11 levels in the membrane fraction and decreased Slc7a11 levels in the cytoplasmic fraction. However, when *Sptbn2* was knocked down in combination with *Hnrnpa2b1* overexpression, Slc7a11 levels in the membrane fraction decreased significantly while those in the cytoplasmic fraction increased. More importantly, total Slc7a11 protein levels remained unchanged throughout (Figure 3I).

### 3.6 Bard1 Knockdown Downregulates Hnrnpa2b1 Ubiquitination Under OGD to Restore *Sptbn2*

*Hnrnpa2b1* was not differentially expressed in the GSE178997, GSE122709, and GSE140275 datasets, suggesting that reduced Hnrnpa2b1 protein may be driven less by transcriptional changes than by post‐translational modifications. Interestingly, our analysis of the GPS‐Uber database (https://gpsuber.biocuckoo.cn/index.php) using the protein sequence of Hnrnpa2b1 (NP_001361674.1) revealed multiple ubiquitination sites on Hnrnpa2b1 (Figure S4F). We predicted the E3 ubiquitin ligases of Hnrnpa2b1 using the UbiBrowser 2.0 database (http://ubibrowser.bio‐it.cn/ubibrowser_v3/home/index). The top 20 E3 ubiquitin ligases (Figure S4G) were intersected with IS‐related genes downloaded from GeneCards. There are five intersections (Figure S4H): Fbh1, Xiap, Bard1, Neurl1, and Pml.

**FIGURE 4:**
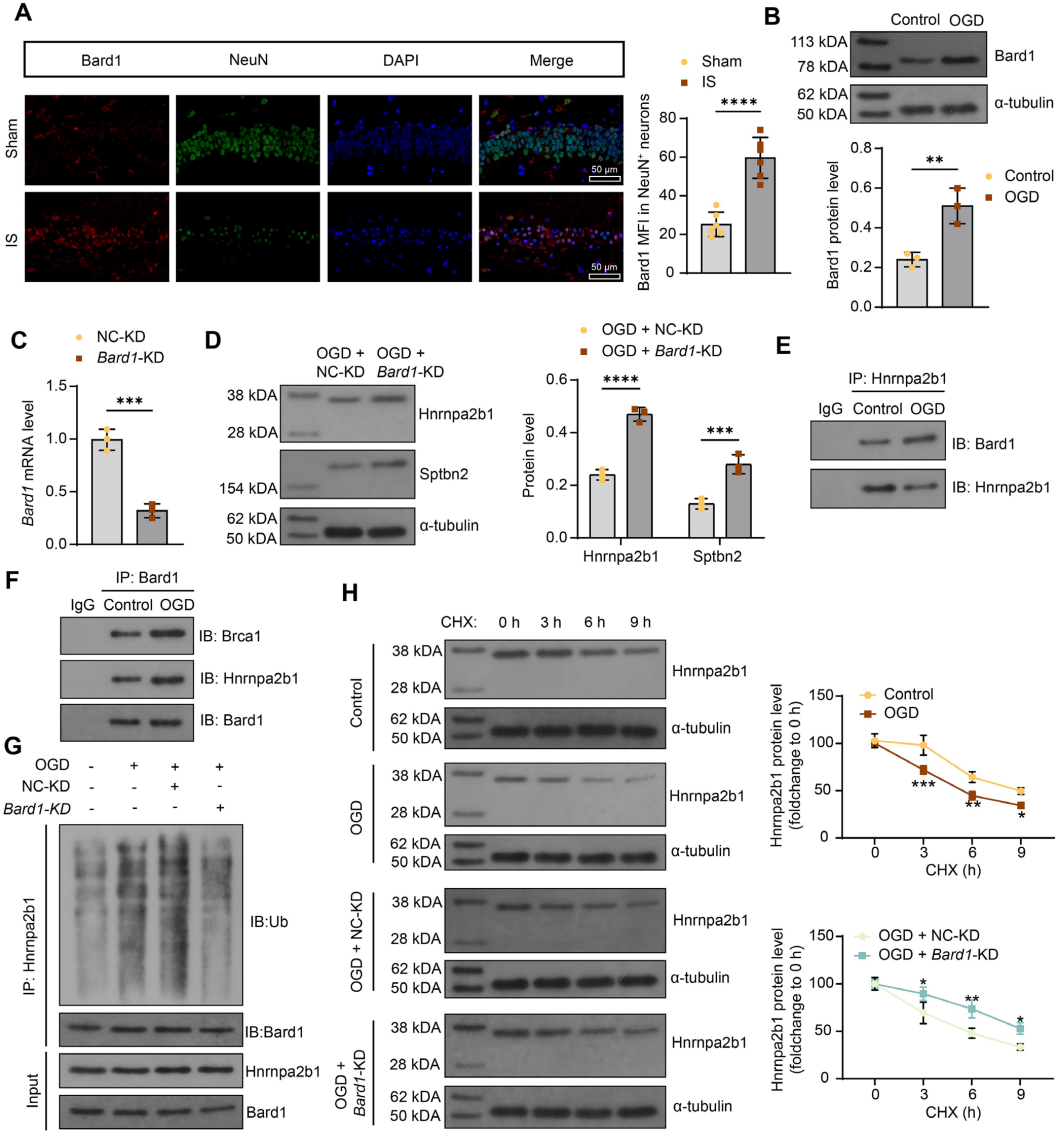
*Bard1* knockdown downregulates Hnrnpa2b1 ubiquitination under OGD conditions to restore Sptbn2 expression. (A) The MFI of Bard1 in hippocampal neurons of the CA1 region of hippocampal tissue of mice was detected by dual‐labeling immunofluorescence (*n* = 6 independent biological replicates). (B) The protein expression of Bard1 in HT‐22 cells with OGD was examined using Western blot assays (*n* = 3 independent biological replicates). (C) *Bard1* expression after *Bard1* knockdown in HT‐22 cells was examined using RT‐qPCR (*n* = 3 independent biological replicates). (D) The protein expression of Hnrnpa2b1 and Sptbn2 after the knockdown of *Bard1* in HT‐22 cells was examined using a Western blot assay (*n* = 3 independent biological replicates). (E) The interaction between Bard1 and Hnrnpa2b1 proteins in HT‐22 cells was analyzed using a Co‐IP assay (*n* = 3 independent biological replicates). (F) The enrichment of Brca1 and Hnrnpa2b1 in Bard1 immunoprecipitation complexes in HT‐22 cells treated with control and OGD was analyzed using a Co‐IP assay (*n* = 3 independent biological replicates). (G) The effect of OGD treatment and knockdown of *Bard1* on ubiquitination modification of Hnrnpa2b1 in HT‐22 cells was examined using a ubiquitination immunoprecipitation assay (*n* = 3 independent biological replicates). (H) The protein stability of Hnrnpa2b1 after OGD and *Bard1* knockdown was examined using a protein half‐life assay (*n* = 3 independent biological replicates). Results are expressed as mean ± SD; * indicates *p* < 0.05; ** indicates *p* < 0.01; *** indicates *p* < 0.001; **** indicates *p* < 0.0001. A two‐tailed unpaired *t*‐test (A–C) and two‐way ANOVA (D, H) were used for statistical analysis, respectively.

Dual‐labeling immunofluorescence revealed a significant increase in the MFI of Bard1 in NeuN‐positive neurons of the CA1 region of the hippocampus in IS mice (Figure 4A). Western blot results confirmed the significant increase in Bard1 expression in HT‐22 cells induced by OGD (Figure 4B). *Bard1* was knocked down in HT‐22 cells (Figure 4C), and knockdown of *Bard1* induced the protein expression of both Hnrnpa2b1 and Sptbn2 in HT‐22 cells (Figure 4D). Co‐IP analysis showed that Bard1 and Hnrnpa2b1 could be detected in the same immunoprecipitation complex (Figure 4E). We further examined the interaction between Bard1 and Brca1. Brca1 was detectable in Bard1 immunoprecipitation complexes, and Brca1 was more highly enriched in Bard1 immunoprecipitation complexes following OGD treatment. Simultaneously, Hnrnpa2b1 was also detectable in Bard1 immunoprecipitation complexes (Figure 4F).

OGD treatment increased Hnrnpa2b1 ubiquitination, while *Bard1* knockdown reduced Hnrnpa2b1 ubiquitination under OGD conditions (Figure 4G). Furthermore, *Bard1* knockdown slowed the degradation of Hnrnpa2b1 under OGD (Figure 4H).

### 3.7 Knockdown of *Bard1* in Mouse Hippocampal Neurons Alleviates PSCI

**FIGURE 5:**
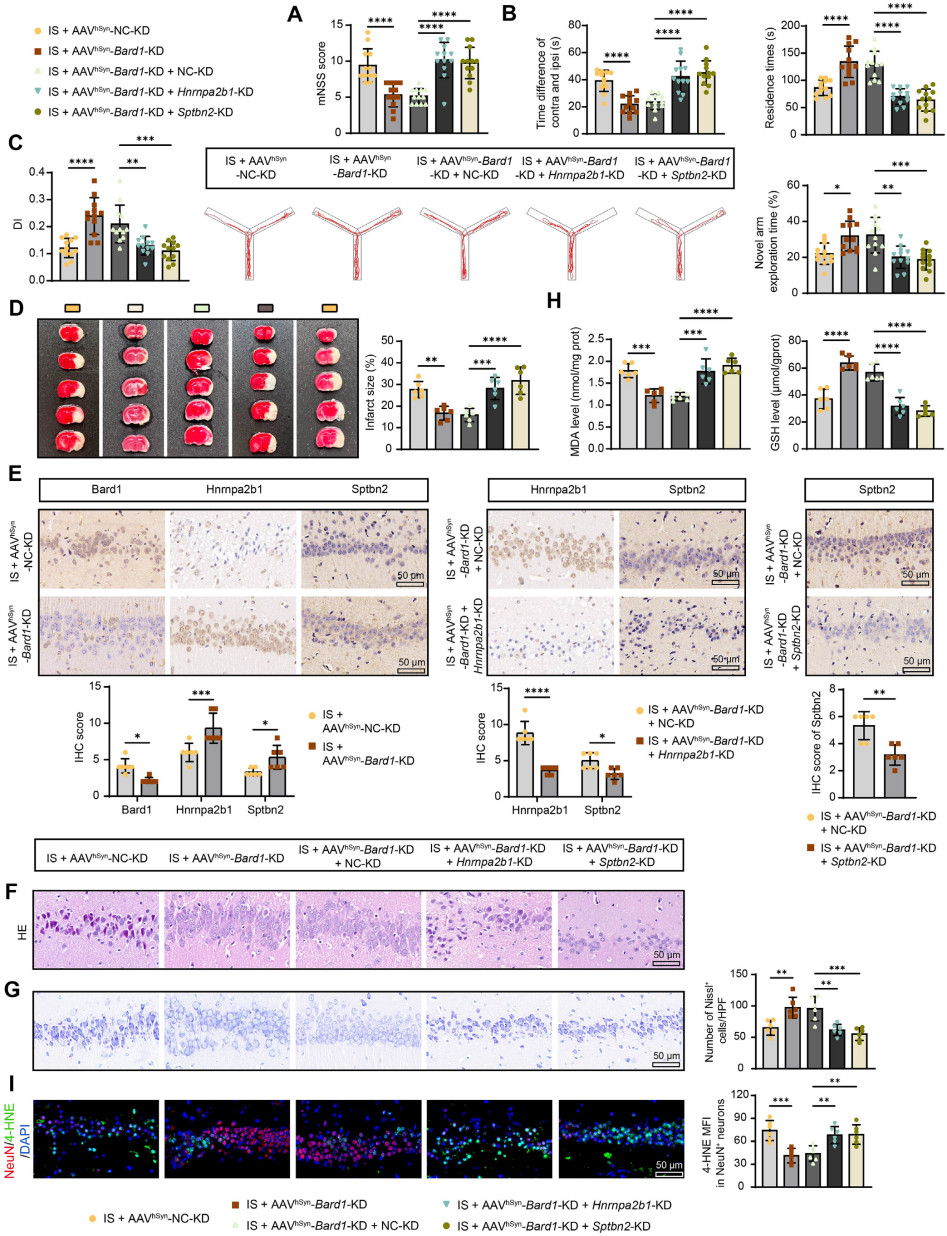
Knockdown of *Bard1* in mouse hippocampal neurons alleviates PSCI in mice. (A) Postoperative neurologic impairment assessed by mNSS scoring (*n* = 12 independent biological replicates). (B) The adhesive removal time between the ipsilateral and contralateral regions of the infarct in the adhesive removal test and the residence time of the mice on the rotarod in the rotarod test (*n* = 12 independent biological replicates). (C) DI in the novel object recognition and novel arm exploration time in the Y‐maze tests (*n* = 12 independent biological replicates). (D) The area of cerebral infarction [(area of the contralateral hemisphere—area of the ipsilateral non‐infarct region)/area of the contralateral hemisphere × 100%] of mice was evaluated using TTC staining (*n* = 6 independent biological replicates). (E) Expression of Bard1, Hnrnpa2b1, and Sptbn2 in hippocampal neurons of the CA1 region of hippocampal tissue of mice was detected by IHC (*n* = 6 independent biological replicates). (F) Neuropathological damage in the CA1 region of the hippocampal tissue of mice was detected by HE staining (*n* = 6 independent biological replicates). (G) The number of Nissl‐positive neurons in the CA1 area of hippocampal tissue of mice was detected by Nissl staining (*n* = 6 independent biological replicates). (H) MDA and GSH content in the hippocampal tissues of mice 24 h after pMCAO was detected by biochemical analysis (normalized to total protein concentration) (*n* = 6 independent biological replicates). (I) The MFI of 4‐HNE in NeuN‐positive neurons in the hippocampal CA1 region of mice in each group 24 h after pMCAO was detected by dual‐label immunofluorescence (*n* = 6 independent biological replicates). Results are expressed as mean ± SD; * indicates *p* < 0.05; ** indicates *p* < 0.01; *** indicates *p* < 0.001; **** indicates *p* < 0.0001. A two‐tailed unpaired *t*‐test (E, the right panel), one‐way ANOVA (A–D, G–I), and two‐way ANOVA (E, the left and middle panels) were used for statistical analysis, respectively.

Bard1‐KD was performed or in combination with *Hnrnpa2b1* (*Bard1*‐KD + *Hnrnpa2b1*‐KD) or *Sptbn2* knockdown (*Bard1*‐KD + *Sptbn2*‐KD) using AAV vectors. IS mice with *Bard1*‐KD had reduced mNSS scores (Figure 5A), partial recovery of functional limb behaviors, and increased dwell time in the rotarod test (Figure 5B), increased DI in the novel object recognition experiment, and prolonged dwell time on the new arm in the Y‐maze (Figure 5C). After *Hnrnpa2b1*‐KD or *Sptbn2*‐KD, mice showed exacerbated neurological impairments and cognitive dysfunction. The increased infarct size corroborated the reversal of the infarct‐relieving effect of *Bard1*‐KD by *Hnrnpa2b1*‐KD or *Sptbn2*‐KD (Figure 5D). IHC confirmed that Bard1 was reduced, whereas Hnrnpa2b1 and Sptbn2 were significantly increased in the CA1 region of the mouse hippocampus after *Bard1*‐KD. Knockdown of *Hnrnpa2b1* in this region decreased the expression of Hnrnpa2b1 and Sptbn2 (Figure 5E). Neuronal degeneration (Figure 5F) and damage (Figure 5G) in the CA1 region of the hippocampus were improved after *Bard1*‐KD, while *Hnrnpa2b1*‐KD or *Sptbn2*‐KD exacerbated hippocampal neuronal damage. Biochemical analysis of hippocampal tissue 24 h post‐surgery revealed that *Bard1*‐KD reduced MDA levels and restored GSH levels. In contrast, MDA levels increased, and GSH levels decreased following the combined *Hnrnpa2b1*‐KD or *Sptbn2*‐KD (Figure 5H). Lastly, *Bard1*‐KD reduced the MFI of 4‐HNE in NeuN‐positive neurons in the CA1 region of the hippocampus in IS mice; however, when *Hnrnpa2b1* or *Sptbn2* was knocked down in combination with *Bard1* knockdown, the MFI of 4‐HNE increased again (Figure 5I).

### 3.8 Knockdown of *Bard1* in HT‐22 Cells Alleviates OGD‐Induced Ferroptosis

**FIGURE 6:**
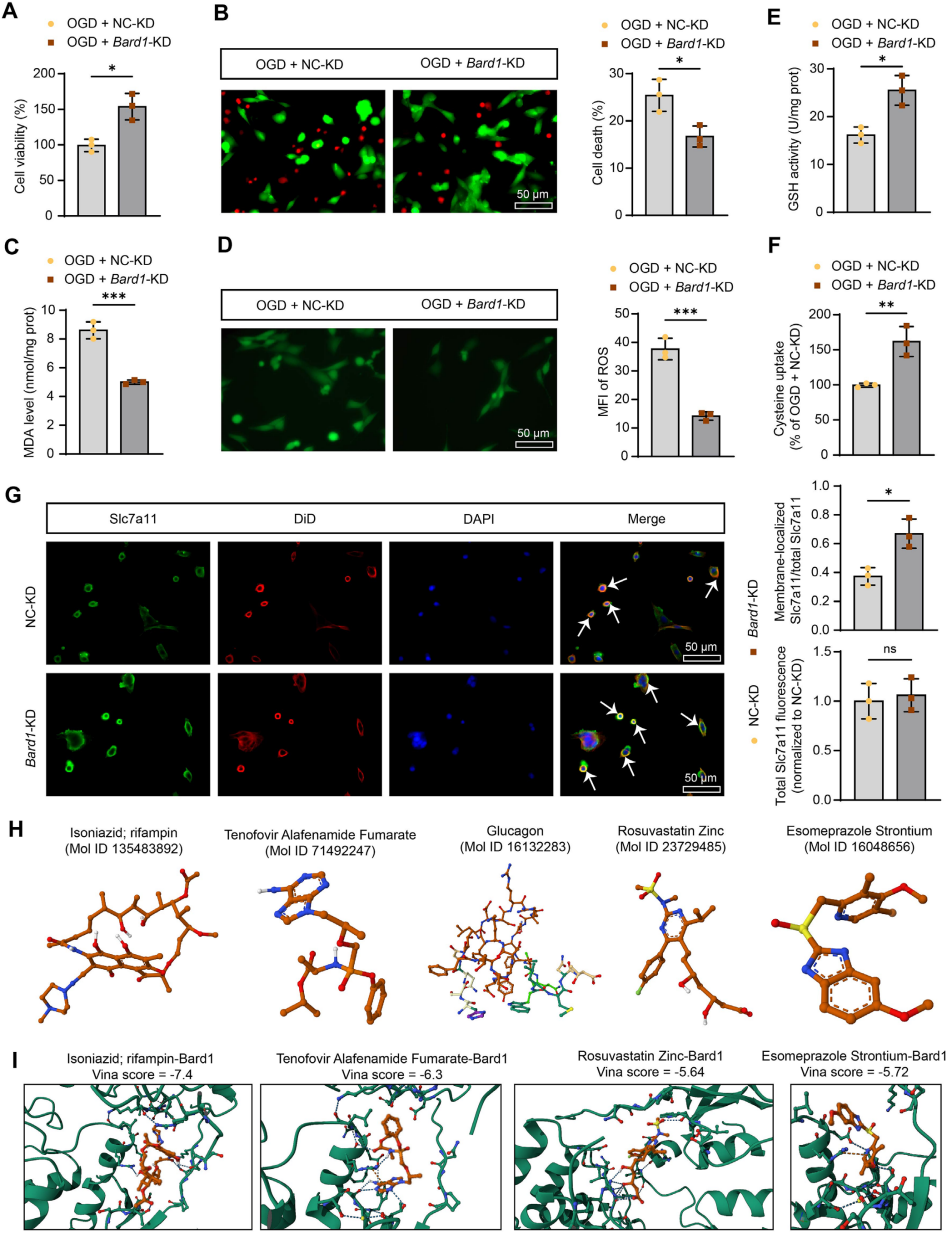
Knockdown of *Bard1* in HT‐22 alleviates OGD‐induced ferroptosis. (A) Cell viability in HT‐22 cells was examined using the CCK‐8 assay. (B) Cell death in HT‐22 cells was examined using live‐dead cell staining. (C) MDA content in HT‐22 cells was examined using an MDA content assay kit. (D) ROS production in HT‐22 cells was examined using a DCFH‐DA probe. (E) GSH content in HT‐22 cells was examined using a GSH assay kit. (F) The cystine uptake capacity of HT‐22 cells was examined using a cystine uptake assay. (G) The membrane transport of Slc7a11 in HT‐22 cells was examined using DiD staining combined with immunofluorescence analysis. The DiD‐positive regions of the cell membrane were designated as regions of interest (ROIs) for membrane localization, and the Slc7a11 fluorescence intensity within this ROI and the total Slc7a11 fluorescence intensity in the same cell were measured. The total Slc7a11 fluorescence intensity was also quantified. (H) Structural formulas and specific details of the top five drug molecules ranked by virtual screening scores. (I) AutoDock Vina docking analysis of the binding of isoniazid; rifampin, tenofovir alafenamide fumarate, rosuvastatin zinc, and esomeprazole strontium with Bard1. *n* = 3 independent biological replicates. Results are expressed as mean ± SD; * indicates *p* < 0.05; ** indicates *p* < 0.01; *** indicates *p* < 0.001. Unpaired *t*‐test (A–G) was used for statistical analysis.

We assessed ferroptosis induced by OGD in HT‐22 cells transfected with either NC‐KD or *Bard1*‐KD. Knockdown of *Bard1* alleviated the OGD‐induced reduction in cell viability (Figure 6A) and increased cell death (Figure 6B). The production of MDA (Figure 6C) and ROS (Figure 6D) in the cells was reduced, GSH activity was increased (Figure 6E), and the uptake capacity of the cells for cystine was partially restored (Figure 6F) following *Bard1*‐KD. After *Bard1*‐KD, membrane expression of Slc7a11 was increased, while the total Slc7a11 fluorescence intensity did not change (Figure 6G).

### 3.9 Identification of Potential Small‐Molecule Candidates Targeting Bard1 via Virtual Screening

To identify novel therapeutic approaches targeting Bard1, we conducted a high‐throughput virtual screening analysis of FDA‐approved drugs with potential Bard1‐targeting activity based on a Bard1 model constructed from the Swiss‐Model database (https://swissmodel.expasy.org/). The top five drugs with the highest predicted scores were selected for analysis (Figure 6H). Using AutoDock Vina for molecular docking, all five drugs showed varying degrees of binding to Bard1 (Figure 6I), except for glucagon, which failed to dock as a large protein. A review of the relevant literature indicates that among the four drug compounds, isoniazid and rifampin, as components of the medication Isoniazid and Rifampicin Tablets, are commonly used for treating pulmonary tuberculosis caused by *Mycobacterium tuberculosis* [16]. Tenofovir alafenamide fumarate is an antiretroviral drug used as a first‐line treatment for HIV and HBV infections [17, 18]. Rosuvastatin zinc, containing rosuvastatin, has demonstrated significant therapeutic effects and potential in stroke‐related diseases [19]. Esomeprazole strontium contains esomeprazole, a novel proton‐pump inhibitor used to treat gastric disorders [20]. Interestingly, esomeprazole has been reported to improve myocardial ischemia–reperfusion injury [21]. It is worth exploring whether it holds similar therapeutic potential in IS, another type of cerebrovascular disease.

## 4 Discussion

IS constitutes approximately 87% of all stroke cases and has emerged as a substantial medical and social challenge because of its close association with cognitive impairment [22]. PSCI severely impacts patients' quality of life and survival [23]. In this study, we identified that *Sptbn2* overexpression reversed neuronal ferroptosis‐related damage via the membrane transport of Slc7a11.

Spectrin dysfunction is implicated in neurodegenerative diseases, heart diseases, and cancers [24]. Although SPTBN2 mutations have been linked to non‐progressive congenital ataxia with marked cognitive impairment in patients [25], the mechanism remains unknown. IS disrupts intracellular iron regulation, contributing to excessive iron accumulation [26]. Our first observation was that OGD‐induced cell damage was comparable to erastin‐induced injury, which was relieved by *Sptbn2* overexpression. Pharmacological inhibition of Slc7a11 leads to GSH depletion and inactivation of GPX4, thereby causing lipid peroxidation‐mediated ferroptosis [27]. Here, we found that ferroptosis in HT‐22 cells induced by the inhibition of GPX4 was not rescued by the upregulation of *Sptbn2*, indicating that the mechanism through which *Sptbn2* regulates ferroptosis is Slc7a11‐dependent. Further, in vivo evidence showed that the infarction area and cognitive impairment were mitigated in mice overexpressing *Sptbn2*. Interestingly, the m^7^G modification of *Sptbn2* mRNA by Mettl1 enhanced its stability and translation, which promoted neurogenesis in Alzheimer's disease [28]. Therefore, we sought to decipher the upstream modifiers of *Sptbn2* in IS.

Hnrnpa2b1 expression was decreased in the hippocampus of rats with bilateral common carotid artery stenosis compared to that of controls [29]. As for its function, the downregulated Hnrnpa2b1 reduced the binding of TrkB m^6^A and promoted TrkB degradation, resulting in low BDNF expression and cognitive impairments [30]. Concurrently, we identified the downregulation of Hnrnpa2b1 in the hippocampal neurons of mice with PSCI and OGD‐induced HT‐22 cells. m^6^A reader fragile X mental retardation protein interacted with splicing factor HnRNPM to recognize the splice site and modulate the exon‐skipping splicing event of the SLC7A11 transcript, and the SLC7A11‐S splicing variant effectively promoted ferroptosis resistance in breast cancer cells [31], indicating the possible mechanism for the Hnrnp family in regulating ferroptosis. However, there is also a recent study showing the promoting effects of Hnrnpa2b1 on cardiac ferroptosis during myocardial ischemia–reperfusion injury, which was related to its modification of PFN2 mRNA [32]. The paradoxical function has yet to be elucidated and might be related to different microenvironments and targets.

Considering that *Hnrnpa2b1* was not differentially expressed in the three GEO datasets that we included, we anticipated that its downregulation might be related to posttranslational modification and identified Bard1 as the E3 ubiquitin ligase. *Hnrnpa2b1* has also been revealed to be regulated by E3 ubiquitin ligases under different cancer conditions [33, 34]. Here, we found that *Bard1*‐KD was beneficial to the narrowing of the infarction area and recovery of cognitive function by restoring Hnrnpa2b1‐*Sptbn2* expression and rescuing ferroptosis. Our experiments also revealed that Brca1 was detectable in Bard1 immunoprecipitation complexes, with enrichment following OGD treatment, and that Hnrnpa2b1 was also present in Bard1 immunoprecipitates, providing direct evidence that Bard1 interacts with both Brca1 and Hnrnpa2b1 under ischemic conditions. The augmented association of Bard1 with Brca1 following OGD is consistent with the canonical role of the BRCA1‐BARD1 heterodimer, which functions as an E3 ubiquitin ligase in DNA damage response and protein stability [35]. The observed enrichment indicates the potential for enhanced Bard1 engagement with Brca1 in response to ischemic stress. This engagement might influence downstream ubiquitination events, thus modulating stress responses.

In this study, we integrated sequencing data from HT‐22 cells subjected to OGD with patient blood sequencing data to identify molecular overlaps between neuronal injury and systemic responses. It is crucial to emphasize that the biological contexts of these datasets are not fully comparable. Consequently, the observed overlaps should be interpreted as exploratory and hypothesis‐generating, rather than definitive evidence of disease mechanisms. Furthermore, administration of AAV‐mediated intervention before pMCAO resulted in a reduction in infarct size at 24 h. This finding suggests that the treatment produced an acute neuroprotective effect. While our data support the involvement of the pathway in post‐stroke cognitive outcomes, we acknowledge that the observed benefits cannot be ascribed exclusively to PSCI mechanisms. Thirdly, the HT‐22 cell line exhibits a deficiency in functional NMDA and AMPA receptors, resulting in its inability to fully replicate excitotoxic neuronal injury that occurs during the acute phase of IS [36]. Future studies employing receptor‐competent neuronal models or in vivo systems will be necessary to fully delineate the contribution of the Bard1‐Hnrnpa2b1‐Sptbn2 axis to IS pathology. In addition, METTL3‐mediated m^6^A methylation promoted stress granule formation and reduced the apoptosis of injured neurons and cells [37], whereas FTO has been demonstrated to promote post‐stroke neuroprotection through m^6^A demethylation of c‐Jun [38]. These alterations underscore the notion that the equilibrium of m^6^A writers and erasers is modified under ischemic stress, potentially impacting neuronal survival and recovery. In this particular context, our findings implicating Hnrnpa2b1 in the regulation of *Sptbn2* should be regarded as part of a broader m^6^A regulatory network. Further research is necessary to elucidate the manner in which METTL3‐mediated methylation and FTO‐mediated demethylation interact with Hnrnpa2b1 binding to regulate *Sptbn2* expression and neuronal outcomes following ischemia. Finally, although our virtual screening identified several FDA‐approved compounds with potential Bard1‐binding activity, their clinical applicability in IS remains uncertain. Each compound possesses a unique pharmacological profile and safety considerations. For instance, isoniazid has been linked to hepatotoxicity [39]; tenofovir alafenamide fumarate is predominantly indicated for viral infections and may not achieve adequate brain penetration; rosuvastatin, while showing promise in the treatment of vascular disease, necessitates close monitoring for muscle toxicity [40]; and esomeprazole's potential neuroprotective effects remain conjectural. Furthermore, evaluation of blood–brain barrier permeability, neurotoxicity risk, seizure liability, and polypharmacy interactions is imperative before the implementation of any translational applications.

In summary, the present findings indicate that Bard1 is associated with ferroptosis‐related neuronal damage through regulation of the Hnrnpa2b1‐Sptbn2 signaling pathway, thereby contributing to both acute brain infarction and subsequent cognitive outcomes. Therefore, our results should be interpreted as evidence that Bard1 participates in neuronal injury by modulating Hnrnpa2b1 ubiquitination and stability under OGD conditions, which may in turn influence cognitive performance.

## Author Contributions

Tuming Li: Writing – review and editing, Writing – original draft, Methodology, Formal analysis. Tianyi Rong: Writing – review and editing, Writing – original draft, Methodology, Formal analysis. Zuoting Shen: Methodology, Formal analysis. Zhenyu Wei: Writing – review and editing, Writing – original draft, Methodology, Formal analysis. Deyan Chen: Data curation, Resources, Validation, Writing – review and editing. Ping Zhong: Formal analysis, Investigation, Software, Visualization, Writing – review and editing. Li Cao: Project administration, Supervision, Validation, Writing – review and editing.

## Funding

This work was supported by the National Natural Science Foundation of China (82371255); the Shanghai Leading Talent Program of Eastern Talent Plan (LJ2025105); the Program for Shanghai Outstanding Academic Leaders (23XD1402500); the Shanghai Science and Technology Innovation Action Plan (23DZ2291500); the Program for Outstanding Medical Academic Leader of Shanghai (2022LJ011); and the Yangpu District Science and Technology Commission and Yangpu District Health Commission Specialized Research Project on Traditional Chinese Medicine (YPZYM202311).

## Ethics Statement

All experiments were conducted under the ethical guidelines of the Animal Experimentation Committee of Shidong Hospital (approval no. 2024–003‐01) and complied with the ARRIVE guidelines.

## Conflicts of Interest

The authors declare no conflicts of interest.

## Supporting information


**Data S1:** Supporting Information.

**Figure S1:** cns71074‐sup‐0002‐FigureS1.docx. *Sptbn2* is a ferroptosis regulator in IS.

**Figure S2:** Fer‐1 partially reverses OGD‐induced neuronal damage exacerbated by *Sptbn2* knockdown.

**Figure S3:** cns71074‐sup‐0004‐FigureS3.docx. *Sptbn2* corrects abnormalities in MDA and GSH levels in hippocampal tissues during the subacute phase following pMCAO.

**Figure S4:** Screening of RBPs regulating *Sptbn2* and E3 ubiquitin ligase regulating Hnrnpa2b1.

**Figure S5:** Hnrnpa2b1 regulates the stability of *Sptbn2*.

**Table S1:** Primer sequences.

**Table S2:** RBPs binding to *Sptbn2* in *Mus musculus* .

## Data Availability

The data that support the findings of this study are available on request from the corresponding author. The data are not publicly available due to privacy or ethical restrictions.
