# Supplementary material for: Bard1‐Mediated Regulation of Hnrnpa2b1 Ubiquitination and Protein Stability Contributes to Neuronal Ferroptosis and Cognitive Dysfunction Following Ischemic Stroke
Source: CNS Neurosci Ther. 2026 Aug 10;32(8):e71074. doi: 10.1002/cns.71074 (PMC13455534; doi:10.1002/cns.71074)
Supplement: Supplementary file 1 [file CNS-32-e71074-s001.docx]

**Figure S3**

**Figure S3** *Sptbn2* corrects abnormalities in MDA and GSH levels in hippocampal tissues during the subacute phase following pMCAO. A. MDA levels in hippocampal tissues from mice at 3, 7, and 14 days after pMCAO were measured by biochemical analysis (normalized to total protein concentration) (n = 6 independent biological replicates). B. GSH levels in hippocampal tissues from mice at 3, 7, and 14 days after pMCAO were measured by biochemical analysis (normalized to total protein concentration) (n = 6 independent biological replicates). Results are expressed as mean ± SD; * indicates *p* < 0.05; ** indicates *p* < 0.01; **** indicates *p* < 0.0001. One-way ANOVA (A-B) was used for statistical analysis.
