# Supplementary material for: Bard1‐Mediated Regulation of Hnrnpa2b1 Ubiquitination and Protein Stability Contributes to Neuronal Ferroptosis and Cognitive Dysfunction Following Ischemic Stroke
Source: CNS Neurosci Ther. 2026 Aug 10;32(8):e71074. doi: 10.1002/cns.71074 (PMC13455534; doi:10.1002/cns.71074)
Supplement: Supplementary file 2 [file CNS-32-e71074-s002.docx]

**Table S1** Primer sequences

| Name of gene | Forward primer sequences (5’-3’) | Reverse primer sequences (5’-3’) |
| --- | --- | --- |
| *Sptbn2* | GTGGCAGAAACACCAGGCATTC | CTCCAGCTTCTCTGACACTACG |
| *Bard1* | ACCATGCTGGATGGACACCGTT | GGCGAGTCATTCTGATAGCCAG |
| *Gapdh* | CATCACTGCCACCCAGAAGACTG | ATGCCAGTGAGCTTCCCGTTCAG |

**Note:** *Sptbn2*, Spectrin beta chain, non-erythrocytic 2; *Bard1*, BRCA1-associated RING domain protein 1; *Gapdh*, glyceraldehyde-3-phosphate dehydrogenase.
