# Supplementary material for: Bard1‐Mediated Regulation of Hnrnpa2b1 Ubiquitination and Protein Stability Contributes to Neuronal Ferroptosis and Cognitive Dysfunction Following Ischemic Stroke
Source: CNS Neurosci Ther. 2026 Aug 10;32(8):e71074. doi: 10.1002/cns.71074 (PMC13455534; doi:10.1002/cns.71074)
Supplement: Supplementary file 3 [file CNS-32-e71074-s003.docx]

**Supplementary methods**

**Acquisition and analysis of public transcriptome datasets**

The relevant data were sourced from the Gene Expression Omnibus (GEO) database, specifically datasets GSE178997, GSE122709, and GSE140275. GSE178997 provides a counts matrix for OGD-treated HT-22 cell RNA-seq data; GSE122709 provides a counts matrix for peripheral blood mononuclear cell RNA-seq data from patients with IS; GSE140275 provides an mRNA fragments per kilobase of exon per million mapped reads (FPKM) matrix for circulating blood RNA-seq data from patients with acute IS. Samples were included based on the source, treatment conditions, and grouping information in the GEO metadata, and only samples with clearly defined groupings that were relevant to the comparative objectives of this study were included. Differential analysis for GSE178997 and GSE122709 was performed using count matrices, while differential analysis for GSE140275 was performed using mRNA FPKM matrices. All results of the differential analysis were corrected for multiple comparisons using the Benjamini-Hochberg method, and genes were selected based on an adjusted *p*-value < 0.01 and |log_2_FC| > 1. Each dataset was analyzed independently, and expression matrices were not merged across datasets. The three datasets were intersected only at the level of differentially expressed gene lists for the screening of candidate molecules.

**Animals and ethics statement**

Eight-week-old C57BL/6 male mice weighing about 22 g were obtained from Shanghai Model Organisms Center, Inc. (Shanghai, China) and kept at 23 ± 1°C under a 12 h light/dark cycle with free food and water access. All experiments were conducted under the ethical guidelines of the Animal Experimentation Committee of Shidong Hospital (approval number: 2024-003-01) and complied with the Guide for the Care and Use of Laboratory Animals.

**Stroke surgery**

After one week of acclimatization, pMCAO surgery was performed. The mice were anesthetized with 2% isoflurane inhalation and placed on a heating blanket, and body temperature was continuously detected via the rectum and maintained at 37°C. We then created a transverse incision between the mouse's eye and ear to expose the zygomatic arch, resected a small segment of the zygomatic arch, and performed a drill opening in the skull to expose the middle cerebral artery. The right middle cerebral artery was permanently ligated using a nylon thread [1]. After suturing the incision, the mice were placed on a heating blanket until they fully recovered. Laser Doppler flowmetry was used to measure cerebral blood flow to ensure occlusion (a reduction of over 80% from baseline values). In this research project, the mouse modeling success rate was approximately 87%. During surgery, about 10% of the mice died directly and were excluded from the study. Mice in the sham-operated group underwent the same surgical maneuver but were not ligated.

Mice were allocated into 9 groups, with at least 24 mice guaranteed to be included in each group. To meet the requirements for tissue sampling and behavioral assessments at different time points, each group of mice was divided into three experimental subgroups. In the first subgroup, 6 mice per group were euthanized 24 h post-surgery, and their brains were harvested for TTC staining to assess the area of cerebral infarction. In the second subgroup, 6 mice per group were euthanized 24 h post-surgery, and their tissues were harvested for histological staining or molecular analysis. The remaining 12 mice in each group served as the behavioral assessment cohort. Approximately 24 h post-surgery, on the same assessment day, they sequentially underwent modified neurological severity scores (mNSS), the adhesive removal test, and the rotarod test. They were maintained until 28 days post-surgery for the novel object recognition test and the Y-maze test. To further analyze the dynamic changes in biochemical markers associated with ferroptosis during the subacute phase, four additional time-course cohorts were established: Sham, IS, IS + AAV^hSyn^-negative control (NC)-overexpression (OV), and IS + AAV^hSyn^-Sptbn2-OV. Tissue samples were collected at 3, 7, and 14 days post-surgery, with six mice per group at each time point, to measure MDA and GSH levels in hippocampal tissues. The mice were euthanized by intraperitoneal injection of 150 mg/kg sodium pentobarbital.

**Intraventricular injection**

To specifically perform genetic intervention in mouse neurons, we obtained adeno-associated viral vectors with neuron-specific promoter hSyn encapsulating overexpression of *Sptbn2*, knockdown of *Bard1*, knockdown of *Hnrnpa2b1*, and knockdown of the *Sptbn2* plasmids from Hanbio (Shanghai, China). At 2 weeks before pMCAO [2, 3], mice were immobilized on a brain stereotaxic apparatus, and the scalp was incised along the midline to mark the fontanel. Viruses were injected into the CA1 region of the hippocampus at a rate of 0.2 μL/min using a micro-syringe with the following injection locations, relative to bregma: -2.7 mm anteroposterior, +3.2 mm mediolateral, and 2.7 mm dorsoventral [4].

**mNSS**

Neurological impairment in mice was assessed by mNSS scoring 24 h after pMCAO. The score included tests of motor, sensory, reflexes, and balance, with a normal state score of 0 and a maximal deficit score of 14. mNSS testing was performed by blinded independent examiners [5].

**Adhesive removal test**

To assess forepaw locomotor deficits (removal time) in each group of mice, three days before ischemia, mice were placed in a 60 × 80 cm opaque plastic box for 60 se for accustom. Next, 24 h after pMCAO, a 6.5-mm-diameter circular adhesive sticker was sequentially applied to the hairless mid-paw region of each right and left forelimb of the animal. The mice were placed back in the opaque box, and the time taken to remove the sticker was recorded. A maximum duration of 120 s was given to the mouse. Each mouse was subjected to 3 trials and given a short rest between trials. Finally, the mean value of the difference in removal time between the ipsilateral and contralateral forepaw in three trials was calculated [6].

**Rotarod test**

The rotarod test was used to assess postoperative motor coordination and balance in mice. Three days before pMCAO, we trained the mice for 3 days, three times a day. Mice were placed on an accelerated rotarod. At the beginning of training, the rotarod speed was accelerated from 8 rpm to 40 rpm, and the residence time of the mice on the rotarod was recorded during the maximum test time of 300 s. At 24 h after pMCAO, the dwell time on the rotarod was recorded as well [7].

**Novel object recognition test**

Twenty-eight days after pMCAO, the mice were placed in the center of the open-field device and allowed to move freely to familiarize themselves with the environment. Each mouse was given two identical objects and placed on the same side of the device. The mice were allotted 10 min to explore and become familiar with the objects, which were identical for all subjects. Before the commencement of the experiment, the device and objects were meticulously cleaned with 70% ethanol. After 1 h, the familiar objects were replaced with novel objects, and the mice were positioned at the center of the device. The time spent exploring each object with the paw or nose within a 2-centimeter area was recorded for five minutes. The discrimination index (DI) was calculated based on these data. The DI is defined as the ratio of time spent exploring new objects to the total time spent.

**Y-maze test**

Twenty-eight days after pMCAO, the Y-maze test was used to analyze the ability of mice to explore and perceive unknown regions. The device is a symmetrically closed 3-armed maze with an angle of 120° between the arms, an arm length of 28 cm, a width of about 5 cm, and a wall height of about 10 cm. In the first phase, one arm was closed off, and each mouse was placed in the center of the apparatus to freely explore the remaining two familiar arms for 10 min. The second phase of the experiment began 1 h later, when the third arm, which had previously been closed off, was opened as a new arm. The mice were then placed back in the center of the apparatus and allowed to freely explore all three arms for 5 min. The time spent exploring the new arms was recorded to assess their spatial recognition memory.

**TTC staining**

At 24 h after surgery, 6 mice in each group were selected for infarct area evaluation. The mice were euthanized with 150 mg/kg sodium pentobarbital (*i.p*), and brain tissues were harvested to make 2-mm-thick coronal cryosections, followed by immersion in 1% TTC staining solution (DK0004, Beijing Leagene Biotech Co., Ltd., Beijing, China) in the dark and fixation with 4% paraformaldehyde. The infarct area (%) was calculated according to the formula: (Area of the contralateral hemisphere – area of the ipsilateral non-infarct region)/area of the contralateral hemisphere × 100%.

**Hematoxylin-eosin (HE) and Nissl staining**

After the remaining 6 mice were also euthanized with 150 mg/kg sodium pentobarbital, the brain tissues were removed. One part was stored at -80°C, and the other part was fixed overnight with formalin and paraffin-embedded to prepare coronal sections (5 μm thick). The sections were stained using an HE staining solution (DH0105, Leagene), and the pathological damage in the CA1 region was observed.

Paraffin-embedded sections of mouse brain tissues from each group were routinely deparaffinized and stained using a Nissl staining solution (PH1211, Phygene, Fuzhou, Fujian, China). The sections were stained in methylene blue staining solution at 37°C for 10 min, treated with Nissl staining for 2 min, with ammonium molybdate solution for 3 min, dehydrated in anhydrous ethanol, and routinely cleared with xylene. The sections were sealed with neutral gum before they were viewed. The number of positive Nissl neurons was counted under the high-magnification field of view in the CA1 region of the hippocampus.

**Immunohistochemistry (IHC)**

Paraffin-embedded sections of mouse brain tissues were dewaxed, rehydrated, and heated to boiling in citrate buffer. The sections were treated with 3% hydrogen peroxide for 10 min, sealed in PBS containing 10% goat serum for 1 h, and probed with primary antibodies to Sptbn2 (1:150, 55107-1-AP, ProteinTech Group, Chicago, IL, USA), Hnrnpa2b1 (1:100, ab283592, Abcam, Cambridge, MA, USA), and Bard1 (1:100, PA5-85707, Thermo Fisher Scientific Inc., Waltham, MA, USA) at 4℃ overnight and with HRP-coupled goat anti-rabbit secondary antibody (1:500, GTX26721, GeneTex, Inc., Alton Pkwy Irvine, CA, USA) at room temperature (RT) for 2 h. After DAB color development, the cell nuclei were counterstained with hematoxylin. The sections were dehydrated with anhydrous ethanol, cleared with xylene, and mounted with neutral gum. Photographs were taken, and observations were made under a microscope. Fields of view were randomly selected from the CA1 region of the hippocampus of each mouse for scoring.

Staining intensity and number of positive cells were used to calculate the IHC score. The degree of staining was meticulously categorized as follows: 0 (no staining), 1 (weak staining), 2 (moderate staining), and 3 (strong staining). The number of positive cells was similarly categorized as 0 (< 5%), 1 (5-25%), 2 (26-50%), 3 (51-75%), and 4 (> 75%). The arithmetic product of the two scores constituted the final score.

**OGD**

HT-22 cells (SNL-202) were procured from Sunncell (Wuhan, Hubei, China) and cultured in DMEM with 10% FBS + 1% P/S at 37°C and 5% CO_2_. For OGD modeling, glucose deprivation was achieved using glucose-free 1× Earle's Balanced Salt Solution (GL0066, Biolab, Beijing, China) incubation, along with a 90-min treatment with 2 mM Na_2_S_2_O_4_ (S433998, Aladdin, Shanghai, China) [8]. For the induction of intracellular ferroptosis, we treated HT-22 cells with 1 μM RSL3 (HY-100218A, MedChemExpress, Monmouth Junction, NJ, USA) or 0.5 μM Erastin (HY-15763, MedChemExpress), respectively, for 24 h. For treatment with a ferroptosis inhibitor, the cells were treated with 1 μM Ferrostatin-1 (Fer-1, HY-100579, MedChemExpress) for 24 h. The control group was treated with equal doses of DMSO.

**Cell transfection**

For genetic intervention in HT-22 cells, we obtained overexpression plasmids or shRNAs from Hanbio. HT-22 cells were collected and cultured in the medium until 80% confluence, and plasmids or shRNAs were transfected into the cells using Lipofectamine 3000. The transfection efficiency was assessed by Western blot analysis or RT-qPCR after 24 h of transfection.

**Cell viability assay**

The treated HT-22 cells were pre-cultured and incubated at 37°C, 5% CO_2_ for 2 h. Next, the CCK-8 solution (B34302, Selleck, Houston, TX, USA) was added and mixed thoroughly, and the cells were incubated for 2 h. The OD value was read at 450 nm, and the cell viability was assessed by the formula cell viability (%) = [OD(treatment group)-OD(blank)]/[OD(control group)-OD(blank)] × 100% to assess cell damage.

**Live-dead cell staining**

Calcein-AM/PI Cell Activity and Cytotoxicity Assay Kit (PH1978, Phygene) was used to analyze cell death. The treated HT-22 cells were treated with 100 μL of staining solution for 15 min at 37°C in the dark, washed with PBS, resuspended, and seeded onto slides to observe the living and dead cells under a fluorescence microscope. The cell death rate (%) was evaluated.

**DCFH-DA staining**

The treated HT-22 cells were cultured on sterile coverslips and treated with 100 μL of DCFH-DA working solution (D6883, Sigma-Aldrich Chemical Company, St Louis, MO, USA) for 10 min. After removing the working solution, the ROS fluorescence was observed. The mean fluorescence intensity was analyzed using ImageJ.

**Cystine uptake assay**

According to the instructions of the Cystine Uptake Assay Kit (UP05, Dojindo Laboratories, Kumamoto, Japan), the treated HT-22 cells were seeded into 96-well plates and cultured overnight. After the removal of the supernatant was removed, the medium was refreshed with serum-free medium without cystine for 5 min, and the cells were incubated with CA Uptake solution at 37℃, 5% CO_2_ in an incubator for 30 min. After the supernatant was discarded, the cells were incubated with 50 μL of methanol and the working solution, and left to stand for 30 min at 37℃, 5% CO_2_ in the incubator. Finally, fluorescence was detected at λex = 490 nm and λem = 535 nm.

**Biochemical analysis**

Malondialdehyde (MDA) content assay kit (D799761, Sangon, Shanghai, China) and reduced GSH colorimetric assay kit (E-BC-K030-M, Elabscience Biotechnology Co., Ltd., Wuhan, Hubei, China) were used to analyze the MDA and GSH content in mouse brain hippocampus tissues and HT-22 cells. For *in vivo* experiments, mouse hippocampal tissues were rapidly isolated 24 h after pMCAO and at dynamic time points during the subacute phase (3, 7, and 14 d), and tissue homogenates were prepared according to the kit instructions. HT-22 cells were harvested and lysed following appropriate treatment. Total protein concentrations were determined for all samples using the BCA method, and MDA and GSH assay results were normalized based on total protein concentration.

**Immunofluorescence staining**

Treated HT-22 cells were fixed using 4% paraformaldehyde and sealed with 5% BSA for 1 h, followed by overnight incubation at 4°C using rabbit antibody to Slc7a11 (1:100, PA1-16893, Thermo Fisher) and with goat-anti-rabbit secondary antibody, Alexa Fluor 488 (1:50, A-11008, Thermo Fisher) for 2 h at RT in the dark. The cell membrane was labeled using DiD Cell Membrane Labeling Solution (V22887, Thermo Fisher). Finally, membrane transport of Slc7a11 was observed under fluorescence microscopy. Membrane localization and quantification of Slc7a11 were performed using ImageJ software. Using the DiD-positive cell membrane region as the membrane-localized region of interest (ROI), the Slc7a11 fluorescence intensity within that ROI and the total Slc7a11 fluorescence intensity in the same cell were measured, and the ratio of membrane-localized Slc7a11 fluorescence intensity to total Slc7a11 fluorescence intensity was calculated. At least 30 cells were randomly selected from each group for analysis.

For mouse brain tissue, paraffin-embedded sections were deparaffinized, rehydrated, heated in sodium citrate, sealed with 10% goat serum for 1 h, and incubated with primary antibodies. For dual-labeling of Sptbn2, Hnrnpa2b1, or Bard1 with NeuN, the sections were incubated with mouse anti-NeuN (1:50, 66836-1-Ig, ProteinTech Group), rabbit anti-Sptbn2 (1:1000, 55107-1-AP, ProteinTech Group), rabbit anti-Hnrnpa2b1 (1:100, ab283592, Abcam), and rabbit anti-Bard1 (1:100, PA5-85707, Thermo Fisher) at 4°C overnight. For dual staining with 4-Hydroxynonenal (4-HNE) and NeuN, the sections were incubated overnight at 4°C with mouse anti-4-HNE (1:25, AB48506, Abcam) and rabbit anti-NeuN (1:3000, ab177487, Acam), The sections were incubated with goat-anti-mouse IgG (1:50, A-11029, Thermo Fisher) and goat-anti-rabbit IgG (1:50, ab150087, Abcam) at RT in the dark for 2 h. After staining cell nuclei with DAPI, the samples were mounted with an anti-fade mountant, and images of the hippocampal CA1 region were acquired under a fluorescence microscope.

Images from all groups of mouse tissues were acquired using the same acquisition parameters. Using ImageJ, NeuN-positive regions were defined as regions of interest (ROIs), and the mean fluorescence intensity (MFI) of Sptbn2, Hnrnpa2b1, Bard1, or 4-HNE, after background subtraction, was quantified. Three non-overlapping fields of view were selected in the CA1 region of the hippocampus from each mouse, and the mean value was recorded as a single data point.

**RIP**

Magna RIP RNA-Binding Protein Immunoprecipitation Kit (17-700, Sigma-Aldrich) was used to perform RIP-qPCR. Briefly, HT-22 cells were collected, and cell lysis was performed in RIP lysis buffer spiked with protease inhibitors and RNase inhibitors, followed by overnight incubation with anti-Hnrnpa2b1 (1:100, ab183654, Abcam) or rabbit anti-IgG preincubated with Protein A/G magnetic beads at 4°C. After enrichment to obtain immunoprecipitated complexes, proteinase K was supplemented to remove the proteins. The RNA was isolated, and the enrichment of *Sptbn2* was analyzed using RT-qPCR.

**RNA stability assay**

HT-22 cells overexpressing Hnrnpa2b1 were exposed to OGD and treated with 5 μg/mL actinomycin D (S8964, Selleck). The cells were harvested at 0, 3, and 6 h of incubation, respectively, and the expression of *Sptbn2* was detected using RT-qPCR.

**Dual-luciferase assay**

To assess whether the predicted m^6^A site was involved in the post-transcriptional regulation of *Sptbn2* by Hnrnpa2b1, we constructed pGL6-miR luciferase reporter plasmids (D2106, Beyotime, Shanghai, China) containing either the wild-type (WT) sequence of the *Sptbn2* 3’UTR or a sequence with a mutation at the predicted m6A site (Mut). Then, we co-transfected the overexpression Hnrnpa2b1 plasmid with a reporter plasmid into HT-22 cells using Lipofectamine 3000 and analyzed the changes in luciferase activity after 48 h of transfection.

**Co-IP**

After appropriate treatment, HT-22 cells were lysed on ice using non-denaturing NP-40 Lysis Buffer (HY-K1002, MedChemExpress) containing protease inhibitors, and total protein was collected after centrifugation at 4°C. The lysate was incubated with anti-Hnrnpa2b1, anti-Bard1 (NB100-319, Novus Biological Inc., Littleton, CO, USA), or normal rabbit IgG overnight at 4°C, followed by incubation with Protein A/G magnetic beads. After the magnetic beads were thoroughly washed with IP lysis buffer, the samples were boiled with SDS-PAGE loading buffer, and the proteins were eluted. Immunoprecipitation samples were analyzed by Western blot for Bard1 and Hnrnpa2b1 to investigate the protein interactions.

**Ubiquitination-immunoprecipitation assay**

Briefly, HT-22 cells were treated with 25 μM MG132 (HY-13259, MedChemExpress) for 4 h to inhibit protein degradation. HT-22 cells were lysed using SDS lysis buffer (1% SDS, 50 mM Tris-HCl, pH 7.5, 150 mM NaCl) and immediately heated at 95°C for 10 min to fully denature the proteins. The SDS concentration was diluted to below 0.1% using NP-40 lysis buffer, and the samples were centrifuged at 4°C to collect the supernatant. A portion of the lysate was harvested as the input sample, and the remaining lysate was incubated with anti-Hnrnpa2b1 (1:100, ab183654, Abcam) by vortexing at 4°C for 2 h. Protein A/G magnetic beads were used to enrich and precipitate the complex. After elution of proteins, they were resuspended in SDS-PAGE buffer and denatured for Western blot analyses.

**Protein half-life measurement**

HT-22 cells with *Bard1* knockdown were subjected to OGD and treated with 10 μg/mL of cycloheximide (CHX, S7418, Selleck). The cells were collected at 0, 3, 6, and 9 h of incubation, respectively, and assayed for the expression of Hnrnpa2b1 using a Western blot. The grayscale values of the Hnrnpa2b1 blot were first normalized to the grayscale values of the α-tubulin blot in the same sample, and normalized to the corresponding values at 0 h for each group, to calculate the relative remaining protein levels of Hnrnpa2b1 at different time points.

**Western blot analyses**

HT-22 cells were lysed using RIPA lysis buffer and centrifuged to extract total protein, and the BCA method was used to quantify the protein concentration. A total of 20 μg of total cellular proteins was separated on 10% SDS-PAGE and transferred to polyvinylidene fluoride membranes. The membranes were sealed with 5% skim milk for 1 h at RT and incubated with the antibodies to Sptbn2 (1:1000, 55107-1-AP, ProteinTech Group), Hnrnpa2b1 (1:1000, ab283592, Abcam), Bard1 (1:500, A1685, ABclonal, Wuhan, Hubei, China), Brca1 (1:1000, A700-319, Thermo Fisher), and α-tubulin (1:1000, #2144, Cell Signaling Technologies, Beverly, MA, USA) at 4℃ overnight, and incubated with HRP-coupled goat anti-rabbit secondary antibody (1:10,000, GTX26721, GeneTex) for 1 h at RT. Immunoreactive bands were visualized using ECL reagents and quantified using ImageJ software. The grayscale values of the target protein blots were normalized to the grayscale values of the α-tubulin blot in the same sample.

**MeRIP-qPCR**

The Magna MeRIP m^6^A Kit (17-10499, Merck KGaA) was used to detect the enrichment levels of m^6^A modifications in *Sptbn2* mRNA. Total RNA was extracted using TRIzol from HT-22 cells treated with Control and OGD, as well as from hippocampal tissue of Sham and IS mice, and fragmented. With 10% of the RNA sample as the input, immunoprecipitation was performed on the remaining RNA using either the anti-m^6^A or mouse IgG provided in the kit. After purification and reverse transcription of the immunoprecipitated RNA, the enrichment levels of *Sptbn2* mRNA were detected using RT-qPCR. The degree of m^6^A enrichment in *Sptbn2* mRNA was calculated after normalization to the input sample and further normalized to the corresponding control group.

**Membrane/Cytoplasmic protein isolation and Slc7a11 detection**

After the HT-22 cells were treated as appropriate and washed with pre-chilled PBS, membrane and cytosolic proteins were isolated using the Mem-PER Plus Membrane Protein Extraction Kit (89842, Thermo Fisher). Total protein was extracted from a separate sample of cells from the same batch using RIPA lysis buffer to measure total Slc7a11 levels. After quantifying the protein concentrations in each group using the BCA assay, equal volumes of protein were subjected to SDS-PAGE separation and transferred to a membrane. Subsequently, Western blot analysis was performed using the anti-Slc7a11 (1:1000, PA1-16893, Thermo Fisher) to detect Slc7a11 protein expression. Na,K-ATPase (1:1000, 3010, Cell Signaling Technologies) was used as the internal control for the membrane fraction, and α-tubulin was used as the internal control for the cytoplasmic fraction and total proteins.

**References**

1.	Zhang P, Zhang X, Huang Y, Chen J, Shang W, Shi G, et al. Atorvastatin alleviates microglia-mediated neuroinflammation via modulating the microbial composition and the intestinal barrier function in ischemic stroke mice. Free Radic Biol Med. 2021; 162:104-117.

2.	Feng S, Li J, Liu T, Huang S, Chen X, Liu S, et al. Overexpression of low-density lipoprotein receptor prevents neurotoxic polarization of astrocytes via inhibiting NLRP3 inflammasome activation in experimental ischemic stroke. Neural Regen Res. 2025; 20(2):491-502.

3.	Wu X, Zheng Y, Liu M, Li Y, Ma S, Tang W, et al. BNIP3L/NIX degradation leads to mitophagy deficiency in ischemic brains. Autophagy. 2021; 17(8):1934-1946.

4.	Yan W, Fan J, Zhang X, Song H, Wan R, Wang W, et al. Decreased neuronal synaptosome associated protein 29 contributes to poststroke cognitive impairment by disrupting presynaptic maintenance. Theranostics. 2021; 11(10):4616-4636.

5.	Zhou H, Yan L, Huang H, Li X, Xia Q, Zheng L, et al. Tat-NTS peptide protects neurons against cerebral ischemia-reperfusion injury via ANXA1 SUMOylation in microglia. Theranostics. 2023; 13(15):5561-5583.

6.	Kolosowska N, Keuters MH, Wojciechowski S, Keksa-Goldsteine V, Laine M, Malm T, et al. Peripheral Administration of IL-13 Induces Anti-inflammatory Microglial/Macrophage Responses and Provides Neuroprotection in Ischemic Stroke. Neurotherapeutics. 2019; 16(4):1304-1319.

7.	Yin D, Wang C, Qi Y, Wang YC, Hagemann N, Mohamud Yusuf A, et al. Neural precursor cell delivery induces acute post-ischemic cerebroprotection, but fails to promote long-term stroke recovery in hyperlipidemic mice due to mechanisms that include pro-inflammatory responses associated with brain hemorrhages. J Neuroinflammation. 2023; 20(1):210.

8.	Wang XJ, Wang MH, Fu XT, Hou YJ, Chen W, Tian DC, et al. Selenocysteine antagonizes oxygen glucose deprivation-induced damage to hippocampal neurons. Neural Regen Res. 2018; 13(8):1433-1439.
