# Supplementary material for: Bard1‐Mediated Regulation of Hnrnpa2b1 Ubiquitination and Protein Stability Contributes to Neuronal Ferroptosis and Cognitive Dysfunction Following Ischemic Stroke
Source: CNS Neurosci Ther. 2026 Aug 10;32(8):e71074. doi: 10.1002/cns.71074 (PMC13455534; doi:10.1002/cns.71074)
Supplement: Supplementary file 4 [file CNS-32-e71074-s004.docx]

**Figure S2**

**Figure S2** Fer-1 partially reverses OGD-induced neuronal damage exacerbated by *Sptbn2* knockdown. A. Sptbn2 expression after knockdown of *Sptbn2* in HT-22 cells was examined using a Western blot assay. B. HT-22 cell viability after knockdown of *Sptbn2* and/or Fer-1 was examined using the CCK-8 assay. C. MDA content in HT-22 cells after knockdown of *Sptbn2* and/or Fer-1 was examined using an MDA content assay kit. D. ROS production in HT-22 cells after knockdown of *Sptbn2* and/or Fer-1 was examined using a DCFH-DA probe. E. GSH content in HT-22 cells with knockdown of *Sptbn2* and/or Fer-1 was examined using a GSH assay kit. n = 3 independent biological replicates. Results are expressed as mean ± SD; * indicates *p* < 0.05; ** indicates *p* < 0.01; *** indicates *p* < 0.001; **** indicates *p* < 0.0001. A two-tailed unpaired t-test (A) and one-way ANOVA (B-E) were used for statistical analysis, respectively.
