# Supplementary material for: Bard1‐Mediated Regulation of Hnrnpa2b1 Ubiquitination and Protein Stability Contributes to Neuronal Ferroptosis and Cognitive Dysfunction Following Ischemic Stroke
Source: CNS Neurosci Ther. 2026 Aug 10;32(8):e71074. doi: 10.1002/cns.71074 (PMC13455534; doi:10.1002/cns.71074)
Supplement: Supplementary file 5 [file CNS-32-e71074-s005.docx]

**Figure S1**

**Figure S1** *Sptbn2* is a ferroptosis regulator in IS. A. The volcano plot of transcriptomics sequencing of OGD-treated HT-22 cells in the GSE178997 dataset. B. The volcano plot of RNA-sequencing of the peripheral blood mononuclear cells of ischemic patients at 24 h and 7 days in the GSE122709 dataset. C. The volcano plot of RNA-sequencing of the peripheral blood mononuclear cells of ischemic patients in the GSE140275 dataset. D. The Venn map of the intersection of differentially expressed genes in the GSE178997, GSE122709, and GSE140275 datasets. E. HT-22 cell viability after RSL3 treatment and overexpression of *Sptbn2* was examined using the CCK-8 assay. F. Cell death in HT-22 cells after RSL3 treatment and overexpression of *Sptbn2* was examined using live-dead cell staining. G. MDA content in HT-22 cells after RSL3 treatment and overexpression of *Sptbn2* was examined using an MDA content assay kit. H. ROS production in HT-22 cells after RSL3 treatment and overexpression of *Sptbn2* was examined using a DCFH-DA probe. n = 3 independent biological replicates. Results are expressed as mean ± SD; ** indicates *p* < 0.01; *** indicates *p* < 0.001; **** indicates *p* < 0.0001. One-way ANOVA (E-H) was used for statistical analysis.
