# Supplementary material for: Bard1‐Mediated Regulation of Hnrnpa2b1 Ubiquitination and Protein Stability Contributes to Neuronal Ferroptosis and Cognitive Dysfunction Following Ischemic Stroke
Source: CNS Neurosci Ther. 2026 Aug 10;32(8):e71074. doi: 10.1002/cns.71074 (PMC13455534; doi:10.1002/cns.71074)
Supplement: Supplementary file 6 [file CNS-32-e71074-s006.docx]

**Figure S4**

**Figure S4** Screening of RBPs regulating *Sptbn2* and E3 ubiquitin ligase regulating Hnrnpa2b1. A. The Venn map of the intersection of IS-related genes in GeneCards and the RBPs regulating *Sptbn2* in the RNAinter database. B. PPI of intersecting genes was developed using the STRING database. C. The potential m^6^A modification sites on the mRNA of *Sptbn2* were predicted using the SRAMP tool. D. RNAinter analysis of interactions between Hnrnpa2b1 and *Sptbn2*. E. The correlation between *HNRNPA2B1* and *SPTBN2* expression in hippocampal tissues was predicted in the GEPIA database. F. Ubiquitination modification sites on the Hnrnpa2b1 protein were predicted in the GPS-Uber tool. G. E3 ubiquitin ligases that regulate Hnrnpa2b1 were predicted in the Ubibrowser2.0 database. H. The Venn map of the intersection of IS-related genes in GeneCards and the E3 ubiquitin ligase that regulates Hnrnpa2b1.
