# Supplementary material for: Bard1‐Mediated Regulation of Hnrnpa2b1 Ubiquitination and Protein Stability Contributes to Neuronal Ferroptosis and Cognitive Dysfunction Following Ischemic Stroke
Source: CNS Neurosci Ther. 2026 Aug 10;32(8):e71074. doi: 10.1002/cns.71074 (PMC13455534; doi:10.1002/cns.71074)
Supplement: Supplementary file 7 [file CNS-32-e71074-s007.docx]

**Figure S5**

**Figure S5** Hnrnpa2b1 regulates the stability of *Sptbn2*. A. The MFI of Hnrnpa2b1 in NeuN-positive neurons of the CA1 region of hippocampal tissue of mice was detected by dual-labeling immunofluorescence (n = 6 independent biological replicates). B. The protein expression of Hnrnpa2b1 in HT-22 cells with OGD was examined using Western blot assays (n = 3 independent biological replicates). C. The m^6^A enrichment levels of *Sptbn2* in hippocampal tissues from Sham and IS mice (normalized to the Input control and further normalized to the Sham group) were examined using MeRIP-qPCR (n = 6 independent biological replicates). D. The m^6^A enrichment levels of *Sptbn2* in Control and OGD-treated HT-22 cells (normalized to the Input and further normalized to the Control group) were examined using MeRIP-qPCR (n = 3 independent biological replicates). E. The protein expression of Hnrnpa2b1 after overexpression of *Hnrnpa2b1* in HT-22 cells was examined using a Western blot assay (n = 3 independent biological replicates). F. *Sptbn2* expression after overexpression of *Hnrnpa2b1* in HT-22 cells was examined using RT-qPCR (n = 3 independent biological replicates). G. The RNA stability of *Sptbn2* after OGD treatment and overexpression of Hnrnpa2b1 in HT-22 cells was examined using an mRNA stability assay (n = 3 independent biological replicates). H. Schematic diagram of luciferase reporter vectors containing the *Sptbn2* 3'-UTR WT sequence or a sequence with a mutation at the predicted m6A modification site (n = 3 independent biological replicates). I. Effect of overexpression of *Hnrnpa2b1* on the activity of WT and Mut vectors was analyzed by luciferase reporter assay (n = 3 independent biological replicates). J. *Sptbn2* mRNA enrichment in the hnrnpa2b1 immunoprecipitation complex was examined using a RIP-qPCR assay (n = 3 independent biological replicates). Results are expressed as mean ± SD; * indicates *p* < 0.05; ** indicates *p* < 0.01; *** indicates *p* < 0.001; and **** indicates *p* < 0.0001. A two-tailed unpaired t-test (A-F, J) and one-way ANOVA (G, I) were used for statistical analysis, respectively.
