# Supplementary material for: Bard1‐Mediated Regulation of Hnrnpa2b1 Ubiquitination and Protein Stability Contributes to Neuronal Ferroptosis and Cognitive Dysfunction Following Ischemic Stroke
Source: CNS Neurosci Ther. 2026 Aug 10;32(8):e71074. doi: 10.1002/cns.71074 (PMC13455534; doi:10.1002/cns.71074)
Supplement: Supplementary file 8 [file CNS-32-e71074-s008.docx]

**Table S2** RBPs binding to *Sptbn2* in *Mus musculus*

| ENOX1 | SF1 |
| --- | --- |
| TARDBP | SRSF1 |
| G3BP2 | SRSF4 |
| IGF2BP2 | ZC3H10 |
| NUPL2 | EIF4B |
| PCBP2 | MBNL1 |
| RBM15B | FUBP1 |
| RBM4B | HNRNPK |
| RBMS3 | NOVA1 |
| SNRNP70 | PCBP1 |
| SRSF11 | PUM1 |
| SRSF9 | RBM45 |
| CELF1 | RBMS2 |
| FMR1 | SFPQ |
| ESRP1 | SRSF10 |
| **HNRNPA2B1** | SRSF5 |
| IGF2BP3 | ZCRB1 |
| PABPC1 | APC |
| PCBP4 | CELF4 |
| RBM24 | SRSF3 |
| RBM5 | PTBP2 |
| SART3 | FUS |
| SNRPA | U2AF2 |
| SRSF2 | RBFOX1 |
| TRA2A | YTHDC2 |
| CELF6 | RBFOX2 |
| LIN28A | TBX5 |
| EWSR1 | SUZ12 |
| HNRNPF | MTF2 |
| KHDRBS3 | EZH2 |
| PABPN1 | CTCF |
| PRR3 | ESRRB |
| RBM25 | JARID2 |
| RBM6 | SPI1 |
